# A number sense as an emergent property of the manipulating brain

**DOI:** 10.1038/s41598-024-56828-2

**Published:** 2024-03-21

**Authors:** Neehar Kondapaneni, Pietro Perona

**Affiliations:** California Institute of Technology, Pasadena, USA

**Keywords:** Human behaviour, Image processing, Network models

## Abstract

The ability to understand and manipulate numbers and quantities emerges during childhood, but the mechanism through which humans acquire and develop this ability is still poorly understood. We explore this question through a model, assuming that the learner is able to pick up and place small objects from, and to, locations of its choosing, and will spontaneously engage in such undirected manipulation. We further assume that the learner’s visual system will monitor the changing arrangements of objects in the scene and will learn to predict the effects of each action by comparing perception with a supervisory signal from the motor system. We model perception using standard deep networks for feature extraction and classification. Our main finding is that, from learning the task of action prediction, an unexpected image representation emerges exhibiting regularities that foreshadow the perception and representation of numbers and quantity. These include distinct categories for zero and the first few natural numbers, a strict ordering of the numbers, and a one-dimensional signal that correlates with numerical quantity. As a result, our model acquires the ability to estimate *numerosity*, i.e. the number of objects in the scene, as well as *subitization*, i.e. the ability to recognize at a glance the exact number of objects in small scenes. Remarkably, subitization and numerosity estimation extrapolate to scenes containing many objects, far beyond the three objects used during training. We conclude that important aspects of a facility with numbers and quantities may be learned with supervision from a simple pre-training task. Our observations suggest that cross-modal learning is a powerful learning mechanism that may be harnessed in artificial intelligence.

## Introduction

### Background

Mathematics, one of the most distinctive expressions of human intelligence, is founded on the ability to reason about abstract entities. We are interested in the question of how humans develop an intuitive facility with numbers and quantities, and how they come to recognize numbers as an abstract property of sets of objects. There is wide agreement that innate mechanisms play a strong role in developing a *number sense*^1–3^, that development and learning also play an important role^2^, that naming numbers is not necessary for the perception of quantities^4,5^, and a number of brain areas are involved in processing numbers^6,7^. Quantity-tuned units have been described in physiology  experiments^3,8–10^ as well as in computational studies^11–14^.

### Related work

The role of learning in developing abilities that relate to the natural numbers and estimation has been recently explored using computational models. Fang et al.^15^ trained a recurrent neural network to count sequentially and Sabathiel et al.^16^ showed that a neural network can be trained to anticipate the actions of a teacher on three counting-related tasks—they find that specific patterns of activity in the network’s units correlate with quantities. The ability to perceive *numerosity*, i.e. a rough estimate of the number of objects in a set, was explored by Stoianov, Zorzi and Testolin^11,12^, who trained a deep network encoder to efficiently reconstruct patterns composed of dots, and found that the network developed units or “neurons” that were coarsely tuned to quantity, and by Nasr et al.^13^, who found the same effect in a deep neural network that was trained on visual object classification, an unrelated task. In these models quantity-sensitive units are an emergent property. In a recent study, Kim et al.^14^ observed that a random network with no training will exhibit quantity-sensitive units. After identifying these units^11–14^, train a supervised classifier on a two-set comparison task to assess numerosity properties encoded by the deep networks. These works showed that training a classifier with supervision, in which the classifier is trained and evaluated on the same task and data distribution, is sufficient for recruiting quantity-tuned units for relative numerosity comparison. Our work focuses on this supervised second stage. Can more be learned with less supervision? We show that a representation for numerosity, that generalizes to several tasks and extrapolates to large quntities, may arise through a simple, supervised pre-training task. In contrast to prior work, our pre-training task only contains scenes with up to 3 objects, and our model generalizes to scenes with up to 30 objects.

### Approach

We focus on the interplay of action and perception as a possible avenue for this to happen. More specifically, we explore whether perception, as it is naturally trained during object manipulation, may develop representations that support a number sense. In order to test this hypothesis we propose a model where perception learns how specific actions modify the world. The model shows that perception develops a representation of the scene which, as an emergent property, can enable the ability to perceive numbers and estimate quantities at a glance^17,18^.

**Figure 1:**
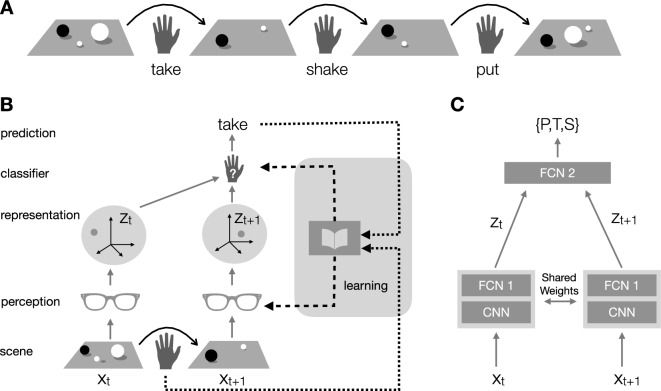
Schematics of our model. (**A**) (Left-to-right) A sequence of actions modifies the visual scene over time. **(B)** (Bottom-to-top) The scene changes as a result of manipulation. The images $x_t$ and $x_{t+1}$ of the scene before and after manipulation are mapped by perception into representations $z_t$ and $z_{t+1}$. These are compared by a classifier to predict which action took place. Learning monitors the error between predicted action and a signal from the motor system representing the actual action, and updates simultaneously the weights of both perception and the classifier to increase prediction accuracy. (**C**) (Bottom-to-top) Our model of perception is a hybrid neural network composed of the concatenation of a convolutional neural network (CNN) with a fully-connected network (FCN 1). The classifier is implemented by a fully connected network (FCN 2) which compares the two representations $z_t$ and $z_{t+1}$. The two perception networks are actually the same network operating on distinct images and therefore their parameters are identical and learned simultaneously in a *Siamese network* configuration ^25^. Details of the models are given in Fig. S15.

In order to ground intuition, consider a child who has learned to pick up objects, one at a time, and let them go at a chosen location. Imagine the child sitting comfortably and playing with small toys (acorns, Legos, sea shells) which may be dropped into a bowl. We will assume that the child has already learned to perform at will, and tell apart, three distinct operations (Fig. 1A). The *put* (P) operation consists of picking up an object from the surrounding space and dropping it into the bowl. The *take* (T) operation consists in doing the opposite: picking up an object from the bowl and discarding it. The *shake* (S) operation consists of agitating the bowl so that the objects inside change their position randomly without falling out. Objects in the bowl may be randomly moved during put and take as well.

We hypothesize that the visual system of the learner is engaged in observing the scene, and its goal is predicting the action that has taken place^19^as a result of manipulation. By comparing its prediction with a copy of the action signal from the motor system it may correct its perception, and improve the accuracy of its predictions over time. Thus, by performing P, T, and S actions in a random sequence, manipulation generates a sequence of labeled two-set comparisons to learn from.

We assume two trainable modules in the visual system: a “perception” module that produces a representation of the scene, and a “classification” module that compares representations and guesses the action (Fig. 1).

During development, perceptual maps emerge, capable of processing various scene properties. These range from basic elements like orientation^20^ and boundaries ^21^ to more complex features such as faces^22^ and objects^23,24^. We propose that, while the child is playing, the visual system is being trained to use one or more such maps to build a representation that facilitates the comparison of the pair of images that are seen before and after a manipulation. These representations are often called embeddings in machine learning.

A classifier network is simultaneously trained to predict the action (P, T, S) from the representation of the pair of images (see Fig. 1). As a result, the visual system is progressively trained through spontaneous play to predict (or, more accurately, post-dict) which operation took place that changed the appearance of the bowl.

**Figure 2:**
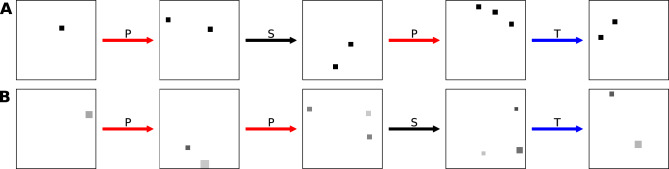
Training image sequence samples. We trained our model using sequences of images that were generated by randomly concatenating take (T), put (P) and shake (S) manipulations, while limiting the number of objects to the $\left\{ 0 \ldots 3 \right\}$ set (see “Methods”-Training Sets). We experimented with two different environment/scene statistics: (**A**) Identical objects ($15\times 15$ pixel squares) with random position. (**B**) Objects (squares) of variable position, size and contrast. The overall image intensity is a poor predictor of cardinality in this dataset (statistics in Fig. S14). Images have been inverted to better highlight objects with low contrast.

We postulate that signals from the motor system are available to the visual system and are used as a supervisory signal (Fig. 1B). Such signals provide information regarding the three actions of put, take and shake and, accordingly, perception may be trained to predict these three actions. Importantly, no explicit signal indicating the number of objects in the scene is available to the visual system at any time.

Using a simple model of this putative mechanism, we find that the image representation that is being learned for classifying actions, simultaneously learns to represent and perceive the first few natural numbers, to place them in the correct order, from zero to one and beyond, as well as estimate the number of objects in the scene.

We use a standard deep learning model of perception^26–28^: a *feature extraction* stage is followed by a *classifier* (Fig. 1). The feature extraction stage maps the image *x* to an internal representation *z*, often called an *embedding*. It is implemented by a deep network^27^ composed of convolutional layers (CNN) followed by fully connected layers (FCN 1). The classifier, implemented with a simple fully connected network (FCN 2), compares the representations $z_t$ and $z_{t+1}$ of the *before* and *after* images to predict which action took place. Feature extraction and classification are trained jointly by minimizing the prediction error. We find that the embedding dimension makes little difference to the performance of the network (Fig. S3). Thus, for ease of visualization, we settled on two dimensions.

We carried out train-test experiments using sequences of synthetic images containing a small number of randomly arranged objects (Fig. 2). When training we limited the top number of objects to three (an arbitrary choice), and each pair of subsequent images was consistent with one of the manipulations (put, take, shake). We ran our experiments twice with different object statistics. In the first dataset the objects were identical squares, in the second they had variable size and contrast. In the following we refer to the model trained on the first dataset as *Model A* and the model trained on the second dataset as *Model B*.

## Results

**Figure 3:**
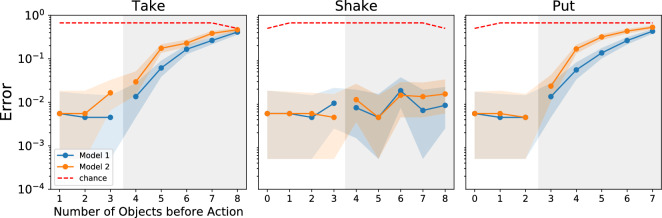
Action classification performance. The network accurately classifies actions up to the training limit of three objects, regardless of the statistics of the data (the x axis indicates the number of objects in the scene before the action takes place). Error increases when the number of objects in the test images exceeds the number of objects in the training set. 95% Bayesian confidence intervals are shown by the shaded areas (272 $\le$ N $\le$ 386). The gray region highlights test cases where the number of objects exceeds the number in the training set. The dashed red line indicates chance level.

We found that models learn to predict the three actions on a test set of novel image sequences (Fig. 3) with an error below 1% on scenes up to three objects (the highest number during training). Performance degrades progressively for higher numbers beyond the training range. Model B’s error rate is higher, consistently with the task being harder. Thus, we find that our model learns to predict actions accurately as one would expect from supervised learning. However, there is little ability to generalize the task to scenes containing previously unseen numbers of objects. Inability to generalize is a well-known shortcoming of supervised machine learning and will become relevant later.

**Figure 4:**
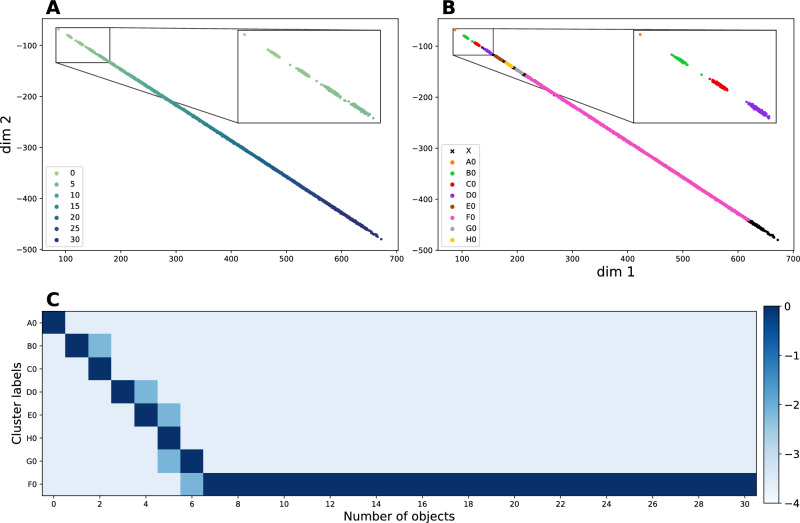
The embedding space for Model B. To explore the structure of the embedding space, we generated a dataset with $\left\{ 0 \ldots 30 \right\}$ objects, extending the number of objects far beyond the limit of 3 objects in the training task. Each image in the dataset was passed through Model B and the output (the internal representation/embedding) of the image is shown. See Fig. S4 for Model A. (**A**) Each dot indicates an image embedding and the embeddings happen to be arranged along a line. The number of objects in each image is color coded. The smooth gradation of the color suggests that the embeddings are arranged monotonically with respect to the number of objects in the corresponding image. The inset shows that the embeddings of the images that contain only a few objects are arranged along the line into “islands”. (**B**) We apply an unsupervised clustering algorithm to the embeddings. Each cluster that is discovered is denoted by a specific color. The cluster X, denoted by black crosses, indicates points that the clustering algorithm excluded as outliers. (**C**) The confusion matrix shows that the clusters that are found by the clustering algorithm correspond to numbers. Images containing 0–6 objects are neatly separated into individual clusters; after that images are collected into a large group that is not in one-to-one correspondence with the number of objects in the image. The color scale is logarithmic (base 10).

When we examined the structure of the embedding we were intrigued to find a number of interesting regularities (Fig. 4). First, the images’ representations do not spread across the embedding, filling the available dimensions, as is usually the case. Rather, they are arranged along a one-dimensional structure. This trait is very robust to extrapolation: after training (with up to three objects), we computed the embedding of novel images that contained up to thirty objects and found that the line-like structure persisted (Fig. 4A). This *embedding line* is also robust with respect to the dimensions of the embedding—we tested from two to 256 and observed it each time (Fig. S3).

**Figure 5:**
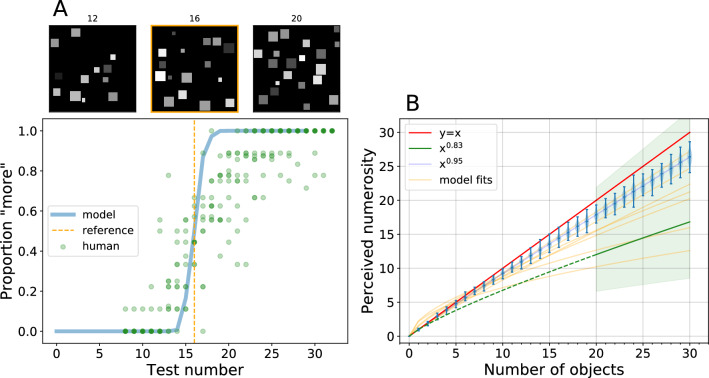
Relative and absolute estimation of quantity. **(A)** Two images may be compared for quantity ^31^ by computing their embedding and observing their position along our model’s embedding line: the image that is furthest along the line is predicted to contain more objects. Here images containing a *test number* of objects (see three examples above containing N = 12, 16 and 20 objects) are compared with images containing the *reference* number of objects (vertical orange dashed line, N = 16). The number of objects in the test image is plotted along the x axis and the proportion of comparisons that result in a “more” response are plotted on the y-axis (blue line). Human data from 10 subjects^32^ is plotted in green. **(B)** The position of images in the embedding space fall along a straight line that starts with 0, and continues monotonically with an increasing number of objects. Thus, the position of an image in the embedding line is an estimate for the number of objects in the scene. Here we demonstrate the outputs of such a model, where we rescale the embedding coordinate (an arbitrary unit) so that one unit of distance matches the distance between the “zero” and the “one” clusters. The y-axis represents such perceived numerosity, which is not necessarily an integer value. The red line indicates perfect prediction. Each violin plot (light blue) indicates the distribution of perceived numerosities for a given ground-truth number of objects. The width of the distributions for the higher counts indicates that perception is subject to errors. There is a slight underestimation bias for higher numbers, consistent with that seen in humans ^33,34^. In fact, Krueger shows that human numerosity judgements (on images with 20–400 objects) follow a power function with an exponent of $0.83 \pm 0.2$. The green line and its shadow depict the range of human numerosity predictions on the same task. The orange lines are power function fits for seven models trained in the same fashion as Model B with different random initializations.

Second, images are arranged almost monotonically along the embedding line according to the number of objects that are present (Fig. 4A). Thus, the representation that is developed by the model contains an order. We were curious as to whether the *embedding coordinate*, i.e. the position of an image along the embedding line, may be used to estimate the number of objects in the image. Any one of the features that make up the coordinates of the embedding provides a handy measure for this position, measured as the distance from the beginning of the line—the value of these coordinates may be thought of as the firing rate of specific neurons^29^. We tested this hypothesis both in a relative and in an absolute quantity estimation task. First, we used the embedding coordinate to compare the number of objects in two different images and assess which is larger, and found very good accuracy (Fig. 5A). Second, assuming that the system may self-calibrate, e.g. by using the “put” action to estimate a unit of increment, then an absolute measure of quantity may be computed from the embedding coordinate. We tested this idea by computing such a *perceived number* against the actual count of objects in images (Fig. 5B). The estimates turn out to be quite accurate, with a slight underestimate that increases as the numbers become larger. Both relative and absolute estimates of quantity were accurate for as many as thirty objects (we did not test beyond this number), which far exceeds the training limit of three. We looked for image properties, other than “number of objects”, that might drive the estimate of quantity and we could not find any convincing candidate (see “Methods” and Fig. S2).

Third, image embeddings separate out into distinct “islands” at one end of the embedding line (Fig. 4A inset). The brain is known to spontaneously cluster perceptual information^7,30^, and therefore we tested empirically whether this form of unsupervised learning may be sufficient to discover distinct categories of images/scenes from their embedding. We found that unsupervised learning successfully discovers the clusters with very few outliers in both Model A and the more challenging Model B (Fig. 4B).

Fourth, the first few clusters discovered by unsupervised learning along the embedding line are in almost perfect one-to-one correspondence with groups of images that share the same number of objects (Fig. 4C). Once such distinct *number categories* are discovered, they may be used to classify images. This is because the model maps the images to the embedding, and the unsupervised clustering algorithm can classify points in the embedding into number categories. Thus, our model learns the ability to carry out instant association of images with a small set of objects with the corresponding number category.

A fifth property of the embedding is that there is a limit to how many distinct number categories are learned. Beyond a certain number of objects one finds large clusters which are no longer number-specific (Fig. 4). I.e. our model learns distinct categories for the numbers between zero and eight, and additional larger categories for, say, “more than a few” and for “many”.

There is nothing magical in the fact that during training we limited the number of objects to three, our findings did not change significantly when we changed the number of objects that are used in training the action classifier (Figs. S6, S7), when we restricted the variability of the objects actions (A.5), and when “put” and “take” could affect multiple objects at once (A.6), i.e. when actions were imprecise. In the last two experiments, we find a small decrease in the separability of clusters in the subitization range (Figs. S9, S12), such that unsupervised clustering is more sensitive to its free parameter (minimum cluster size).

## Discussion

Our model and experiments demonstrate that a representation of the first few natural numbers, absolute numerosity perception, and subitization may be learned by an agent who is able to carry out simple object manipulations. The training task, action prediction, provides supervision for two-set comparisons. This supervision is limited to scenes with up to 3 objects, and yet the model can successfully carry out relative numerosity estimation on scenes with up to 30 objects. Furthermore, action prediction acts as a pretraining task that gives rise to a representation that can support subitization and absolute numerosity estimation without requiring further supervision.

The two mechanisms of the model, deep learning and unsupervised clustering, are computational abstractions of mechanisms that have been documented in the brain.

A number of predictions are suggested by the regularities in the image representation that emerge from our model.

First, the model discovers the structure underlying the integers. The first few numbers, from zero to six, say, emerge as categories from spontaneous clustering of the embeddings of the corresponding images. Clustered topographic numerosity maps observed in human cortex may be viewed as confirming this prediction^7^. These number categories are naturally ordered by their position on the embedding line, a fundamental property of numbers. The ability to think about numbers may be thought of as a necessary, although not sufficient, step towards counting, addition and subtraction^35,36^. The dissociation between familiarity with the first few numbers and the ability to count has been observed in hunter-gatherer societies^5^ suggesting that these are distinct steps in cognition. In addition, we find that these properties emerge even when the number of objects involved in the action is random, further relaxing the assumptions needed for our model (Sec. A.6).

Second, instant classification of the number of objects in the scene is enabled by the emergence of number categories in the embedding, but it is restricted to the first few integers. This predicts a well-known capability of humans, commonly called *subitization*^17,37^.

Third, a linear structure, which we call *embedding line*, where images are ordered according to quantity, is an emergent representation. This prediction is strongly reminiscent of the *mental number line* which has been postulated in the psychology literature^38–41^. The embedding line confers to the model the ability to estimate quantities both in relative comparisons and in absolute judgments. The model predicts the ability to carry out relative estimation, absolute estimation, as well as the tendency to slight underestimation in absolute judgments. These predictions are confirmed in the psychophysics literature^31,33^.

Fourth, subitization and numerosity estimation extend far beyond the number of objects used in training. While the model trains itself to classify actions using up to three objects, subitization extends to 5–8 objects and numerosity estimation extends to at least thirty, which is as far as we tested. Extrapolating from the training set is a hallmark of abstraction, which eludes most supervised models^42^, yet has been shown in rhesus monkeys^43^. Consensus in the deep networks literature is that models *interpolate* their training set, while here we have a striking example of generalization *beyond* the training set.

Fifth, since in our model manipulation teaches perception, one would predict that children who lack the ability or the drive to manipulate would show retardation in the development of a number sense. A study of children with Developmental Coordination Disorder^44^ is consistent with this prediction.

Sixth, our model predicts that adaptation affects estimation, but not subitzation. This is because subitization solely relies on classifiers, which allows for a direct estimate of quantity. Estimation, however, relies on an analog variable, the coordinate along the embedding line, which requires calibration. These predictions are confirmed in the psychophysics literature^31,33^.

Seventh, our model predicts the existence of summation units, which have been documented in the physiology literature ^29^ and have been postulated in previous models^45^. It does not rule out the simultaneous presence of other codes, such as population codes or labeled-line codes ^9^.

The model is simple and our clustering method is essentially *parameter-free*. Our observations are robust with respect to large variations in the dimension of the embedding, the number of objects in the training set and the tuning parameters of the clustering algorithm. Yet, the model accounts qualitatively and, to some extent, quantitatively for a disparate set of observations by psychologists, psychophysicists and cognitive scientists.

There is a debate in the literature on whether estimation and subitization are supported by the same mechanisms or separate ones^31,46^. Our model suggests a solution that supports both arguments: both perceptions rely on a common representation, the embedding. However, the two depend on different mechanisms that take input from this common representation.

It is important to recognize the limitations of our model: it is designed to explore the minimal conditions that are required to learn several cognitive number tasks, and abstracts over the details of a specific implementation in the brain. For instance, we limit the model to vision, while it is known that multiple sensory systems may contribute, including hearing, touch and self-produced actions^47–49^. Furthermore, the visual system serves multiple tasks, such as face processing, object recognition, and navigation. Thus, it is likely that multiple visual maps are simultaneously learned, and it is possible that our “latent representation” is shared with other visual modalities^13^. Additionally, we postulate that visually-guided manipulation, and hence the ability to detect and locate objects, is learned before numbers. Thus, it would perhaps be more realistic to consider input from an intermediate map where objects have been already detected and located, and are thus represented as “tokens”, in visual space, and this would likely make the model’s task easier, perhaps closer to Model A than to Model B. However, making this additional assumption is not necessary for our observations.

An interesting question is whether object manipulation, which in our model acts as the supervisory signal during play, may be learned without supervision and before the learner is able to recognize numbers. Our work sheds no light on this question, and simply postulates that this signal is available and, importantly, that the agent is able to discriminate between the three put, take and shake actions. Our model shows that this simple signal on scenes containing a few objects may be bootstrapped to learn about integers, and to perform subitization and numerosity estimation in scenes containing many objects.

Our investigation adds a concrete case study to the discussion on how abstraction may be learned without explicit supervision. While images containing, say, five objects will look very different from each other, our model discovers a common property, i.e. the number of items, which is not immediately available from the brightness distribution or other scene properties. The mechanism driving such abstraction may be interpreted as an implicit *contrastive learning* signal^50^, where the *shake* action identifies pairs of images that ought to be considered as similar, while the *put* and *take* actions signal pairs of images that ought to be considered dissimilar, hence the clustering. However, there is a crucial difference between our model and traditional contrastive learning. In contrastive learning, the similarity and dissimilarity training signals are pre-defined for each image pair and the loss is designed to achieve an intended learning goal—to bring the embeddings of similar images together and push the embeddings of dissimilar images apart. In our model, image pairs are associated by an action and the network is free to organize the embeddings in any manner that would be efficient for solving the action prediction task. The learned representation is surprisingly robust—while the primary supervised task, action classification, does not generalize well beyond the three objects used in training, the abstractions of number and quantity extend far beyond it.

## Methods

### Network details

The network we train is a standard deep network^28^ composed of two stages. First, a feature extraction network maps the original image of the scene into an embedding space (Fig. 1A). Second, a classification network takes the embedding of two sequential images and predicts the action that modified the first into the second (Fig. 1B). Given the fact that the classification network takes the embedding of two distinct images as its input, each computed by identical copies of the feature extraction network, the latter is trained in a Siamese configuration^25^.

The feature extraction network is a 9-layer CNN followed by two fully connected layers (details in Fig. S15A). The first 3 layers of the feature extraction network are from AlexNet^27^ pre-trained on ImageNet^51^ and are not updated during training. The remaining four convolutional layers and two fully connected layers are trained in our action prediction task.

The dimension of the output of the final layer is a free parameter (it corresponds to the number of features and to the dimension of the embedding space). In a control experiment we varied this dimension from one to 256, and found little difference in the action classification error rates (Fig. S3). We settled for a two-dimensional output for the experiments that are reported here.

The classification network is a two-layer fully connected network that outputs a three-dimensional one-hot-encoding vector indicating a put, take or shake action (details in Fig. S15B).

#### Training procedure

The network was trained with a negative log-likelihood loss (NLL loss) function with a learning rate of 1e-4. The NLL loss calculates error as the -log of the probability of the correct class. Thus, if the probability of the correct class is low (near 0), the error is higher. The network was trained for 30 epochs with 30 mini-batches in each epoch. Each mini-batch was created from a sequence of 180 actions, resulting in 180 image pairs. Thus, the network saw a total of 162,000 unique pairs of images over the course of training.

We tested for reproducibility by training Model B thirty times with different random initializations of the network and different random seeds in our dataset generation algorithm. The embeddings for these reproduced models are shown in Fig. S7.

#### Compute

All models were trained on a GeForce GTX TITAN X using PyTorch. Each model takes at most 20 min to train. We train a total of 106 models (including supplemental experiments).

### Synthetic dataset details

#### Training sets

We carried out experiments using synthetic image sequences where objects were represented by randomly positioned squares. The images were $244\times 244$ pixels (px) in size. Objects were positioned with uniform probability in the image, with the exception that they were not allowed to overlap and a margin of at least 3px clearance between them was imposed. We used two different statistics of object appearance: identical size (15px) and contrast (100%) in the first, and variable size (10–30px) and contrast (9.8–100%) in the second (Fig. 2). Mean image intensity statistics for the two training sets are shown in Fig. S14. The mean image intensity is highly correlated with the number of objects in the first dataset, while it is ambiguous and thus not very informative in the second. We elaborate on covariates like mean image intensity in the following section.

Each training sequence was generated starting from zero objects, and then selecting a random action (put, take, shake) to generate the next image. The take action is meaningless when the scene contains zero objects and was thus not used there. We also discarded put actions when the objects reached a maximum number. This limit was three for most experiments, but limits of five and eight objects were also explored (Fig. S6).

#### Test sets

In different experiments we allowed up to eight objects per image (Fig. 3, S6) and thirty objects per image (Figs. 4, 5A,B) in order to assess whether the network can generalize to tasks on scenes containing previously unseen numbers of objects. The first test set (up to 8 objects) was generated following the same recipe as the training set. The second test (up to 30 objects) set was generated to have random images with the specified number of objects (without using actions), this test set is guaranteed to be balanced. In section A.1, we use the 30 object test set to estimate covariates for numerosity and analyze their impact on task performance. We were unable to find an image property that would “explain away” the abstraction of number (Fig. S2). We note that a principled analysis of the information that is carried out by individual object images is still missing from the literature ^52^ and this point deserves more attention.

### Action classification performance

To visualize how well the model was able to perform the action classification task, we predict actions between pairs of images in our first test set. The error, calculated by comparing the ground truth actions to the predicted actions, is plotted with respect to the number of objects in the visual scene at $x_t$. 95% Bayesian confidence intervals with a uniform prior were computed for each data point, and a lower bound on the number of samples is provided in the figure captions (Figs. 3, S3, S6).

### Interpreting the embedding space

We first explored the structure of the embedding space by visualizing the image embeddings in two dimensions. The points, each one of which corresponds to one image, are not scattered across the embedding. Rather, they are organized into a structure that exhibits five salient features: (a) the images are arranged along a one-dimensional structure, (b) the ordering of the points along the line is (almost) monotonic with respect to the number of objects in the corresponding images, (c) images are separated into groups at one end of the embedding, and these groups are discovered by unsupervised learning, (d) these first few clusters are in one-to-one correspondence with the first few natural numbers, (e) there is a limit to how many number-specific clusters are discovered (Fig. 4).

To verify that the clusters can be recovered by unsupervised learning we applied a standard clustering algorithm, and found almost perfect correspondence between the clusters and the first few natural numbers (Fig. 4). The clustering algorithm used was the default Python implementation of HDBSCAN^53^. HDBSCAN is a hierarchical, density based clustering algorithm, and we used the euclidean distance as an underlying metric^54^. HDBSCAN has one main free parameter, the minimum cluster size, which was set to 90 in Fig. 4. All other free parameters were left at their default values. Varying the minimum cluster size between 5 and 95 does not have an effect on the first few clusters, although it does create variation in the number and size of the later clusters. Beyond 95, the algorithm finds only three clusters corresponding to 0, 1 and greater than 1.

One additional structure is not evident from the the embedding and may be recovered from the action classifier: the connections between pairs of clusters. For any pair of images that are related by a manipulation, two computations will be simultaneously carried out; first, the supervised action classifier in the model will classify the action as either P, T, or S (Fig. 3) and, at the same time, the unsupervised subitization classifier (Fig. S5A) will assign each image in the pair to the corresponding number-specific cluster. As a result, each pair of images that is related by a P action provides a directed link between a pair of clusters (Fig. S5A, red arrows), and following such links one may traverse the sequence of numbers in an ascending order. The T actions provide the same ordering in reverse (blue arrows). Thus, the clusters corresponding to the first few natural numbers are strung together like the beads in a necklace, providing an unambiguous ordering that starts from zero and proceeds through one, two etc. (Fig. S5A,B). The numbers may be visited both in ascending and descending order. As we pointed out earlier, the same organization may be be obtained more simply by recognizing that the clusters are spontaneously arranged along a line, which also supports the natural ordering of the numbers ^40,55,56^. However, the connection between the order of the number concepts, and the actions of put and take, will support counting, sum and subtraction.

To estimate whether the embedding structure is approximately one dimensional and linear in higher dimensions we computed the one-dimensional linear approximation to the embedding line, and measured the average distortion of using such approximation for representing the points. More in detail, we first defined a mean-centered embedding matrix with M points and N dimensions, each point corresponding to the embedding of an image. We then computed the best rank 1 approximation to the data matrix by computing its singular value decomposition (SVD) and zeroing all the singular values beyond the first one. If the embedding is near linear, this rank 1 approximation should be quite similar to the original matrix. To quantify the difference between the original matrix and the approximation, we calculated the element-wise residual (the Frobenius norm of the difference between the original matrix and the approximation), then computed the ratio of the Frobenius norm of the residual matrix and the Frobenius norm of the original matrix. The nearer the ratio is to 0, the smaller the residual, and the better the rank 1 approximation. We call this ratio the *linear approximation error*, we show this error compared to some embeddings in Fig. S7. We computed the embedding for dimensions 8, 16, 64, and 256, (one experiment each) and found ratios of 0.70%, 2.23%, 2.77%, and 2.24%, suggesting that they are close to linear.

### Estimating relative quantity

We can use the perceived numerosity to reproduce a common task performed in human psychophysics. Subjects are asked to compare a reference image to a test image and respond in a two-alternative forced choice paradigm with “more” or “less”. We perform the same task using the magnitude of the embedding as the fiducial signal. The model responds with more if the embedding of the test image has a larger perceived numerosity than the reference image. The psychometric curves generated by our model are presented in Fig. 5A and match qualitatively the available psychophysics ^31,34^.

### Estimating absolute quantity

As described above, the clusters are spaced regularly along a line and the points in the embedding are ordered by the number of objects in the corresponding images (Fig. S5). We postulate that the number of objects in an image is proportional to the distance of that image’s embedding from the embedding of the empty image. Given the linear structure, any one of the embedding features, or their sum, may be used to estimate the position along the embedding line. In order to produce an estimate we use the embedding of the “zero” cluster as the origin. The zero cluster is special, and may be detected as such without supervision, because all its images are identical and thus it collapses to a point. The distance between “zero” and “one”, computed as the pairwise distance between points belonging to the corresponding clusters, provides a natural yardstick. This value, also learned without further supervision, can be used as a unit distance to to interpret the signal between 0 and n. This estimate of numerosity is shown in Fig. 5B against the actual number of objects in the image. We draw two conclusions from this plot. First, our unsupervised model allows an estimate of numerosity that is quite accurate, within 10–15% of the actual number of objects. Second, the model produces a systematic underestimate, similar to what is observed psychophysically in human subjects ^33^.

### Supplementary Information

Supplementary Information.

## Data Availability

All data generated or analysed during this study are included in this published article and its supplementary information files. The data can also be generated with the code.
