# Supplementary material for: A number sense as an emergent property of the manipulating brain
Source: Sci Rep. 2024 Mar 21;14:6858. doi: 10.1038/s41598-024-56828-2 (PMC10958013; doi:10.1038/s41598-024-56828-2)
Supplement: Supplementary file 1 — Supplementary Information. [file 41598_2024_56828_MOESM1_ESM.pdf]

A Number Sense as an Emergent Property of the Manipulating Brain (Supplementary Material)

N. Kondapaneni and P. Perona – California Institute of Technology

March 13, 2024

A Additional Experiments

A.1 Controlling for spurious correlates of "number"

Do image properties, other than the abstraction of "object number", drive the quantity estimate of our model? Many potential *confound variables*, such as the count of pixels that are not black, are correlated with object number and might play a role in the model's ability to estimate the number of objects in the scene. If that were the case, one might argue that our model is not learning the abstraction of "number", but rather learning to measure image properties that are correlated with number.

We controlled for this hypothesis by exploiting the natural variability of our test set images. We explored three image properties that correlate with the number of objects and might thus be exploited to estimate the number of objects: (a) overall image brightness, (b) the area of the envelope of the objects in the image, and (c) the total number of pixels that differ from the background. Since objects in training set B vary both in size and in contrast, these three variables are not deterministically related to object number and thus, we reason, counfound variable fluctuations ought to affect error rates independently of the number of objects.

We focused on close-call relative estimate tasks (e.g. 16 vs 18 objects), where errors are frequent both for our model and for human subjects, and, while holding the number of objects constant in each of the two scenes being compared, we studied the behavior of error rates as a function of fluctuations in the confound variables. One would expect more errors when comparing image pairs where quantities that typically correlate with the number of objects are anticorrelated in the specific example (Fig. S1). Conversely, one would expect lower error rates when the confound variables are positively correlated with number.

In Fig. S2 error rates are plotted vs each one of the confound variables when the n. of objects is held constant. We could not find large systematic biases even for extreme variations in the confound variables. In conclusion, we do not find support for the argument that any of the confound variables we studied is implicated significantly in the estimate of quantity.

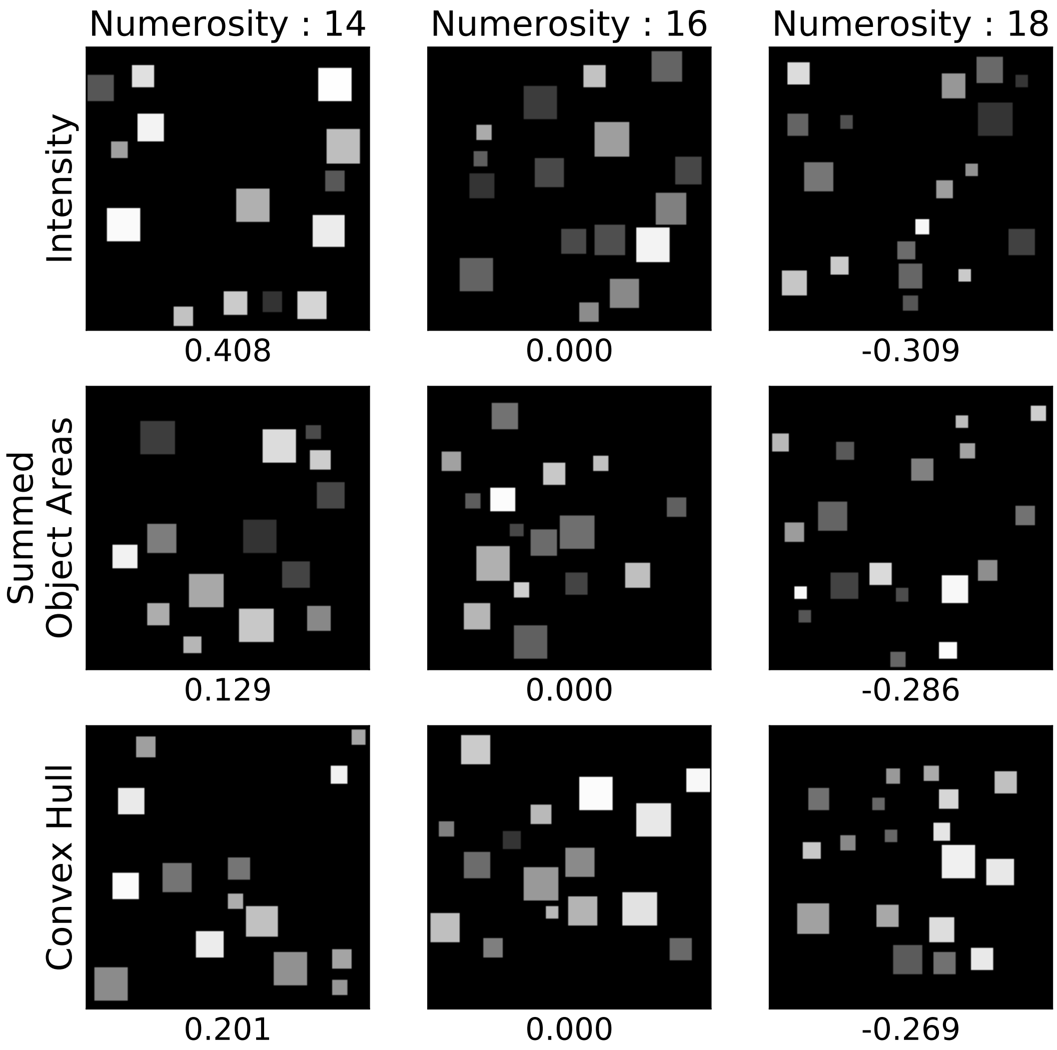

Figure S1: Sample images where covariates are anticorrelated with number. We sample images where the three covariates we study (one covariate per row) are anticorrelated with the number of objects. The number below each plot shows the fractional difference from the value of the covariate in the reference image (center column). For example, in the top right, there is a 30.9% decrease in average image intensity when compared to the intensity in the reference image (center column). Another example: in the last row, the scene with 18 objects has a 26.9% smaller convex hull than the corresponding scenes with 14 and 16 objects. For each row, from the lowest numerosity to the highest, the model predicts a perceived numerosity of 12.82, 14.01, and 16.60 (Intensity); 13.21, 14.43, 15.55 (Summed Object Area); 13.22, 15.28, 16.44 (Convex Hull). Thus, our model correctly classifies the relative numerosity for each one of the image pairs that may be formed from each row (our model slightly underestimates numerosity, see Figure 5B.) Image pairs formed this way are used in the experiments shown in Figure S2, where this manipulation was repeated multiple times and confidence intervals were computed.

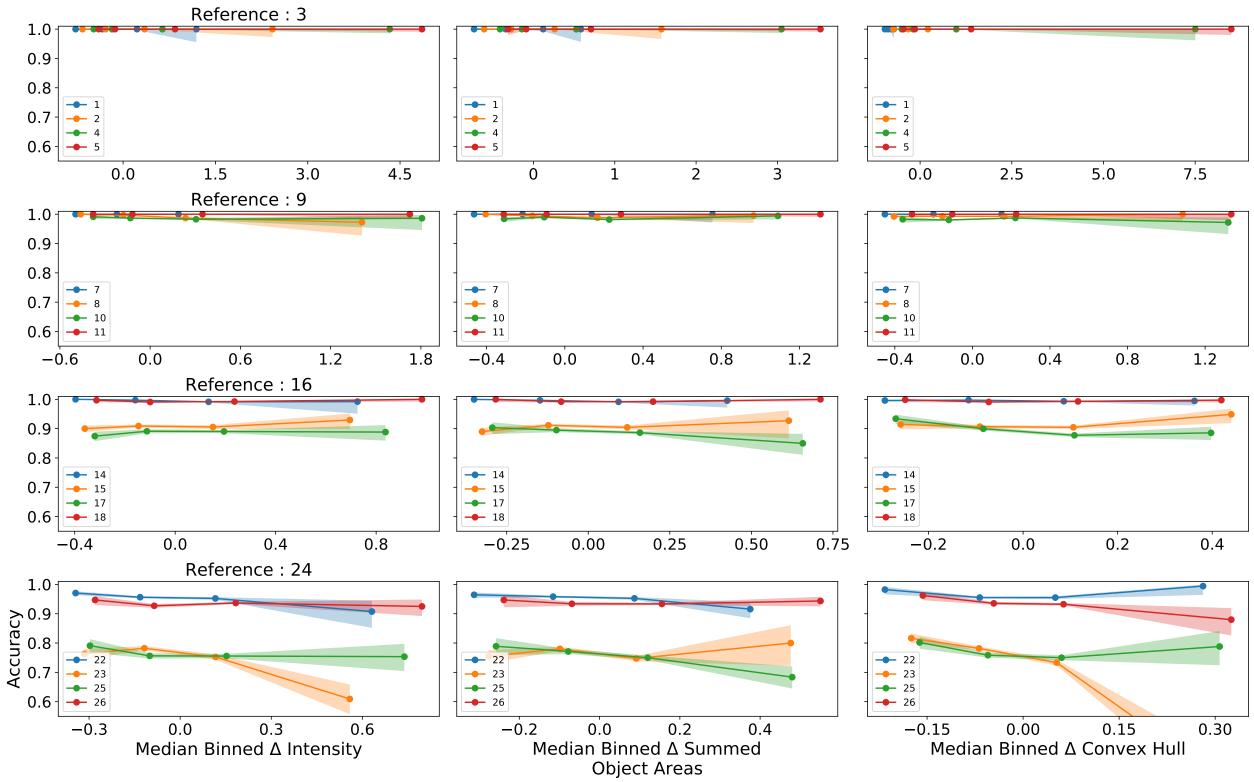

Figure S2: Effects of covariates of numerosity. Three covariates of the number of objects in the scene are explored for possible influence on our model's estimate of numerosity. These are average image intensity **(left column)**, the sum of the areas of the objects **(middle column)**, and the area of the objects' convex hull **(right column)**. Each plot shows the error rates in a relative quantity discrimination task like the one in Figure 5A. We generate a test set of 4650 test images, 150 images per number of objects. For each plot we chose reference images containing respectively 3, 9, 16 and 24 objects (rows of the figure) and had our model judge relative numerosity w.r. to test images containing a different but similar number of objects (indicated in the legend and associated with colors). Given the stochastic nature of the images, the covariates vary over a wide range for each number of objects (see examples in Fig. S1). For each number of objects, we plot the model's error rates (y axis) as a function of the value of the covariate quantity (x axis) which is expressed as fractional difference from the reference image (the values are binned). Shadows display 95% Bayesian confidence intervals($N > 100$, where N is bin size). Horizontal error lines indicate no correlation of numerosity estimation with the covariate quantity. A few lines have slopes that differ slightly from zero indicating a possible correlation. However, some of the slopes indicate a negative correlation (i.e. the better the signal, the higher the error rate). From this evidence it is difficult to conclude that that the model is exploiting anything but "number" to estimate numerosity.

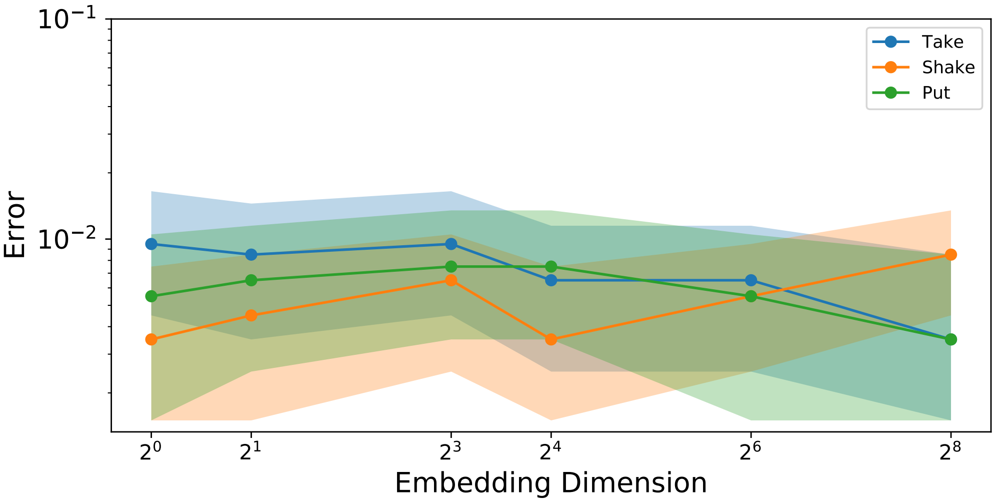

Figure S3: Action classification error as a function of embedding dimension. Classification errors for Model B, averaged over the number of items in the scene (0 - 3) are plotted as a function of the dimension of the embedding (a free parameter in our model). Since the effect is minimal we arbitrarily picked a dimension of two for ease of visualization (Figs. 4, S5). The shadows show 95% Bayesian confidence intervals ($287 \leq N \leq 355$).

A.2 Interpreting the Embedding Space

Does the dimension of the embedding space influence the action classification error? We wondered what is the effect of this free parameter on the model's performance. We explored this question by training our model repeatedly with the same training images, and varying the dimension of the embedding (Fig. 1). Figure S3 shows that the effect of the embedding dimension is negligible. This was initially surprising to us. An explanation may be found in the fact that learning produces an embedding that is organized as a line (see Fig 4 and Sec. A.4).

Next, we explored the structure of the embedding space in the region where images containing 0-3 objects (the training range) are represented. As discussed in the main text we find that the embedding is organized into clusters (Fig. S5 (A,B)). Each cluster contains embeddings of images with the same number of objects. For each pair of images that were generated by a *put* action we drew a red arrow connecting the corresponding embeddings. We used blue arrows for *take* pairs. It is clear from the figure that by following the red arrows one may visit numbers in increasing order: 0-1-2-3 and vice-versa for blue arrows, i.e. the embedding that is produced by our model supports counting up and down.

A.3 Varying Training Limit

In our main experiment we trained our model to classify actions with scenes containing from zero to three objects. Does this choice influence qualitatively or quantitatively our

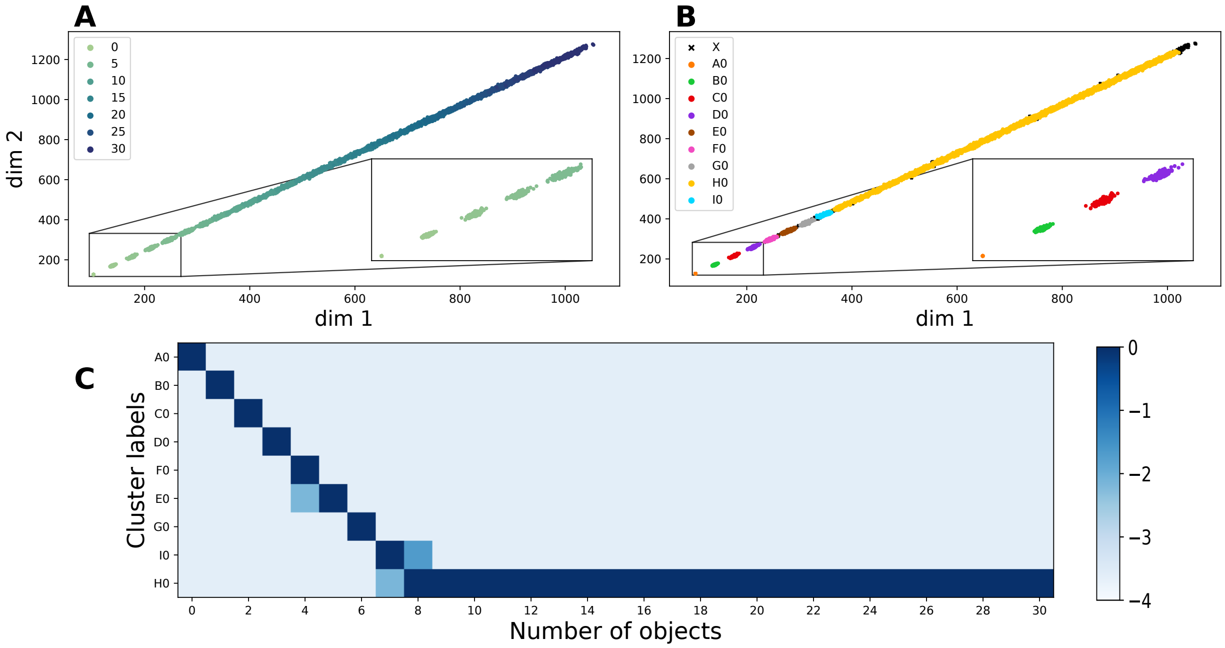

Figure S4: The embedding space for Model A. We reproduce Fig. 4 for model A. **(A)** Similar to Model B, we observe a monotonically increasing line with well seperated groups at lower quantities. **(B)** We apply an unsupervised clustering algorithm to the embeddings. Each cluster that is discovered is denoted by a specific color. The cluster X, denoted by black crosses, indicates points that the clustering algorithm excluded as outliers. **(C)** The confusion matrix shows that the clusters that are found by the clustering algorithm correspond to numbers. Images containing 0 - 7 objects are neatly separated into individual clusters; after that images are collected into a large group that is not in one-to-one correspondence with the number of objects in the image. The color scale is logarithmic (base 10).

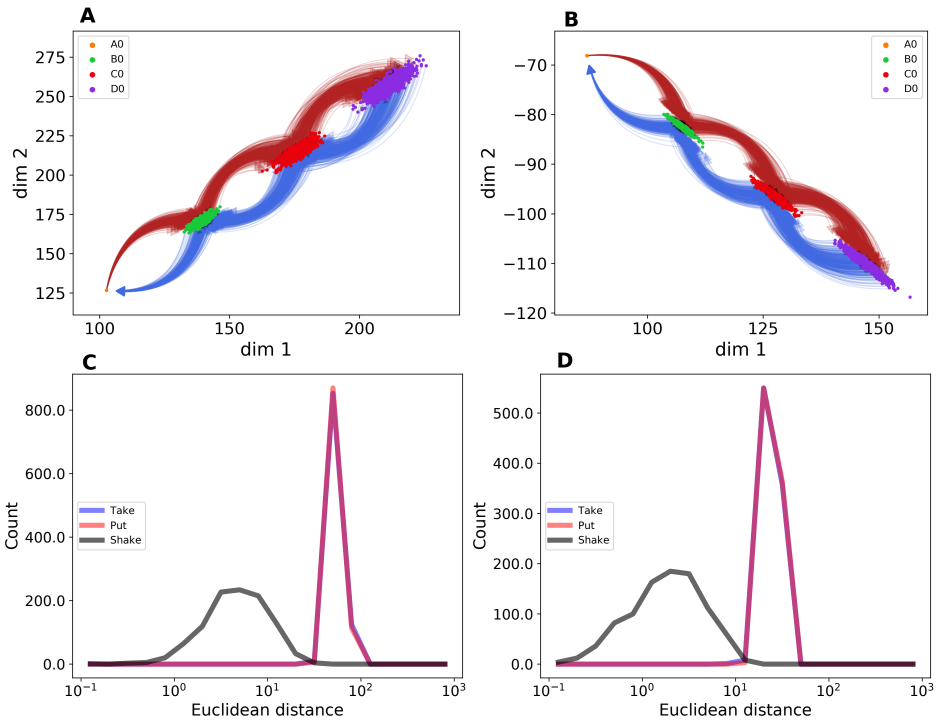

Figure S5: Embeddings with topology for Model A and Model B. A close-up look at the embedding space within the training limit. The left side are plots from Model A and the right side from Model B. **(A), (B)** Unsupervised clustering is performed on the embedding space. Each embedding is colored by it's cluster. Each cluster A0 - D0 correspond to images with numerosities 0 - 3. The clusters are well-separated. The "zero" clusters, for both Model A and Model B, are immediately recognizable as they have no variance (orange dot). As numerosity increases, Model A clusters remain well-separated, whereas Model B clusters begin to come closer to each other. We also overlay a topology from the training actions (P), (T), (S). Blue arrows joining a pair of points represent take actions, red arrows represent put actions. Arrows representing shake actions are under the point clouds and are mostly not visible. **(C), (D)** Distances between pairs of points in the embedding space are histogrammed by action. The histograms show the clearly different distribution for shake actions in comparison to take and put actions. Furthermore, the overlap between shake and non-shake actions is smaller for Model A than Model B, explaining the higher performance in action classification for Model A.

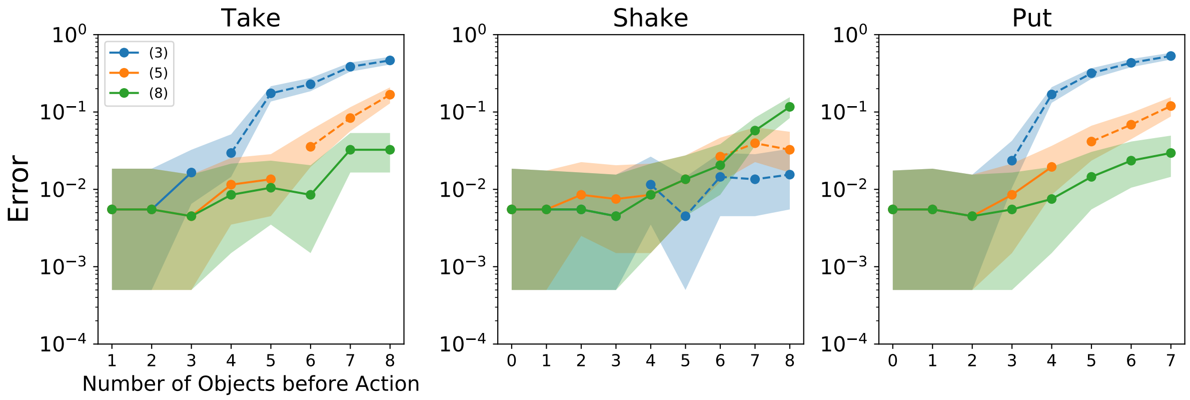

Figure S6: Effect of modifying the training limit. (see also Fig. 3) In order to explore the effect of the number of objects during training, we trained the network to predict actions using a maximum of 3, 5, or 8 objects with images like those in dataset B (Fig. 2B). We tested the network on 8 objects. Each panels shows errors on the training task and are in the same style as Figure 3. The line-breaks and dashed lines mark where the training limit ends and the testing region begins, and the legend shows the training limit in parentheses. The shadows provide 95% confidence intervals ($287 \leq \mathrm{N} \leq 355$). As expected, the error is lower when the training limit is higher.

observations?

To explore this question we re-trained our model using images that were generated with a total number of three, five and eight objects. As expected, we find that adding more objects to the training images reduces the action classification error for image pairs with corresponding number of objects (Fig. S6). We find no change in the linearity of the embeddings, however, the number of clusters seems to increase with the training limit (Figs. S7A,B). This increase in clusters that corresponds with training limit likely explains the improvement in action classification performance.

A.4 Reproducibility of the 1D structure of the embedding

The line-like organization of our embedding space is a striking feature. Is this the result of chance, or is this a robust feature that may be reproduced reliably?

We explored this question by repeating our experiments, varying each the random seed used to generate the training images, as well as the random seed used to initialize the model perception network's weights. We show all the embeddings we obtained in Fig. S7. Each time we measured how line-like are the embeddings and we report the deviation from an exact line as a percent error below each embedding. We found that the deviations from a perfect line are very small, and most look perfectly linear with a few exceptions where we see slight kinks in the line.

A.5 Restricting Dataset Variability

In our main experiment the arrangement of the objects in the scene varied randomly between *put*, *take* and *shake* actions. The size and contrast were varied as well. This was because we did not wish to presume that the agent (a child) playing with the objects would have to be careful with their motions. Furthermore we did not wish to presume that lighting conditions, and thus image contrast, and object pose, and thus their apparent size, would be preserved during the play session. However, one may suspect that scene randomness could help the model abstract the concept of "number" without being distracted by other factors such as object placement, contrast and size.

We explored the effect of randomness by modifying the process that generates data for Model B. In dataset B, object properties (area, intensity) are completely randomized before and after an action (Fig. 2B). We thus constructed a new dataset (Fig. S8), where we restricted the randomness before and after an action by reducing the amount of change in an object's area and intensity to a small amount of jitter. However, we still randomize object position, which we find is fundamental to learning a generalizable model of numerosity. We find that even after reducing object variation, the model has learned has the same properties as Model B (Fig. S9). However, learning is more sensitive to the initial seed (Fig. S10). We refer to this dataset as the *jitter dataset* and model's trained by this dataset as *Jitter Models*.

A.6 Imprecise Action Sizes

Will our model learn the abstraction of "number" even when the *put* and *take* actions will place or remove an unpredictable random number of objects?

We explored this question by randomizing the number of objects that each action affects in the range 0-3, as opposed to exactly 1 as in the main experiment. We capped the maximum number of objects to 3, like previous experiments. We find that while precise actions help in building distinct clusters in the subitization range, it is not necessary to retain the important properties of the generalizable number line. We refer to this dataset as the *imprecise actions dataset* (Fig. S11) and model's trained by this dataset as *Imprecise Action Models*. We find that all the properties of the original model retained (Fig. S12) and that the model is reproducible (Fig. S13).

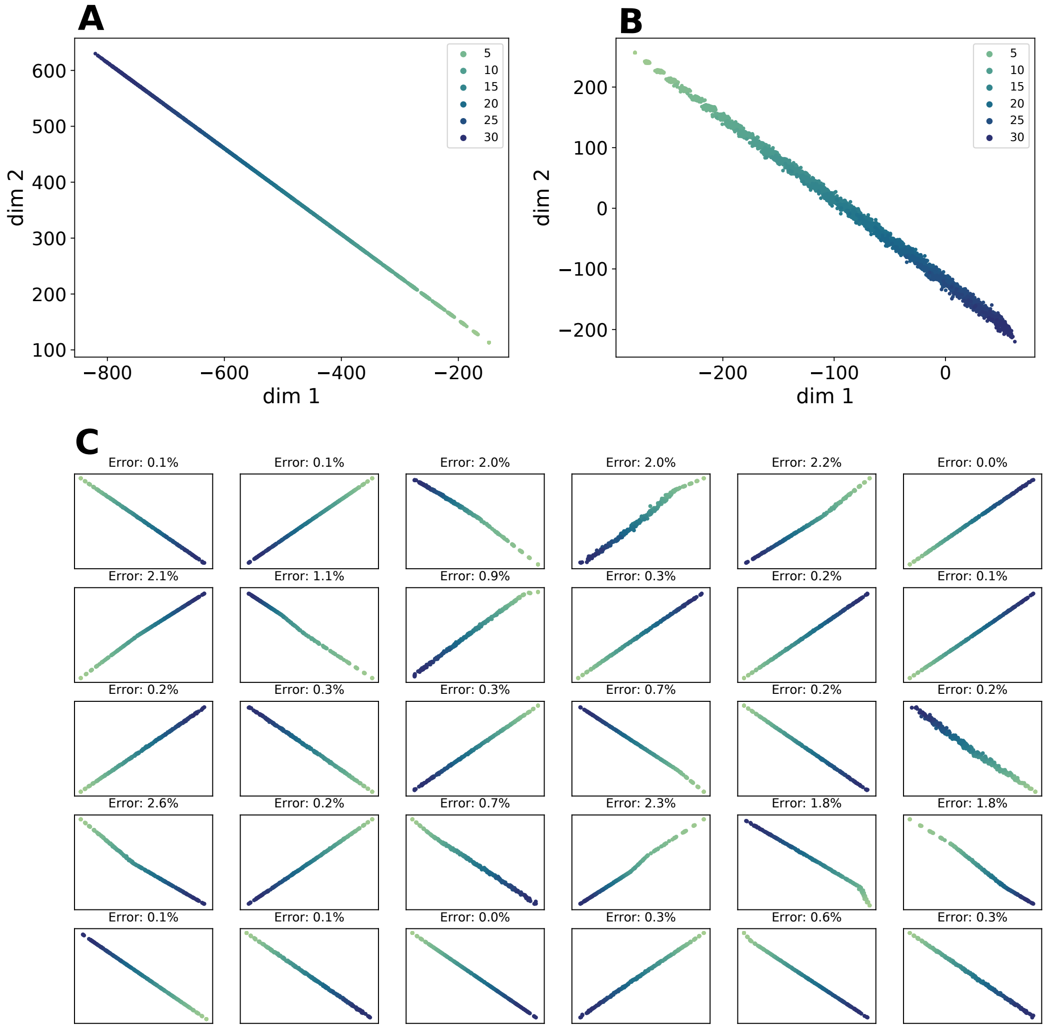

Figure S7: Miscellaneous embedding spaces. (see also Fig. 4) **(A)** Embedding space for the network trained on dataset B, with up to five objects. **(B)** Embedding space for the network trained on dataset B, with up to eight objects. **(C)** Embedding spaces for 30 different random initializations. We repeated the training procedure 30 times on different random initializations of dataset B, with a training limit of 3 objects. Qualitatively, 21 embedding spaces look like a straight line, six initializations present a slight kink in the line, and three instances either present a large kink or two kinks. The linear approximation error (Methods - Interpreting the Embedding Space) is provided above each subplot and measures the approximate deviation from a purely linear model. An error below 4% predicts an approximately linear embedding line.

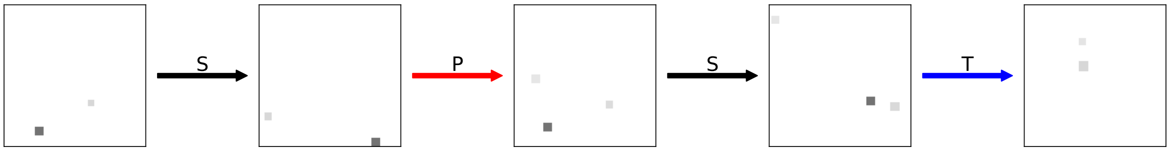

Figure S8: Jitter Datasets. In Jitter Datasets, we restrict the change in size and contrast an object may undergo due to an action. After each action, the size (diagonal) of an object will be allowed to jitter by up to 3 pixels and the contrast by $\pm 0.02\%$ of the maximum contrast. We find that these small perturbations in object representations are sufficient to recreate similar results to those seen with Model B.

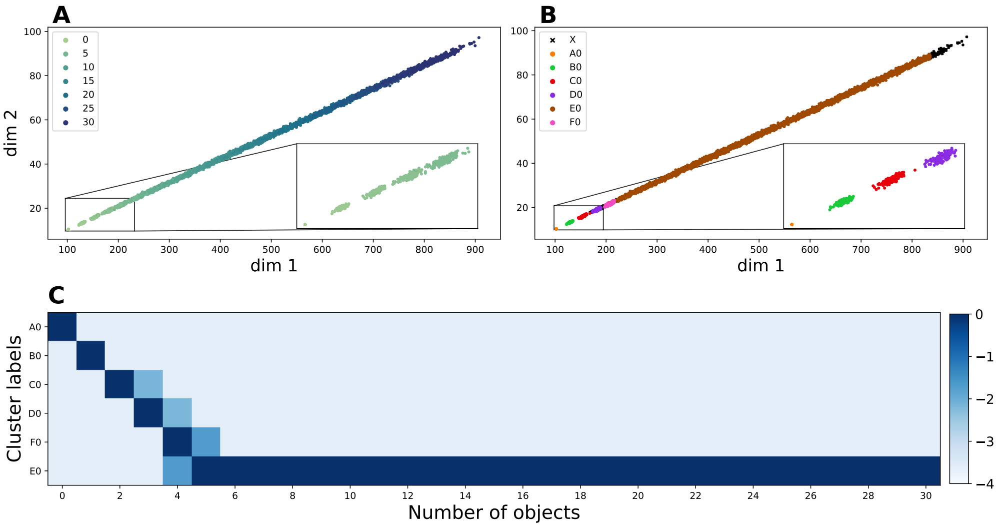

Figure S9: Properties of Jitter Models. We find that the important properties of the Model B representation arise with Jitter Models. The model representations are linear, monotonic, with the early numbers easily separable. We set the minimum cluster size to 30 (HDBSCAN), in order to produce the most concise plots. Note the Jitter Model representations are more sensitive to minimum cluster size.

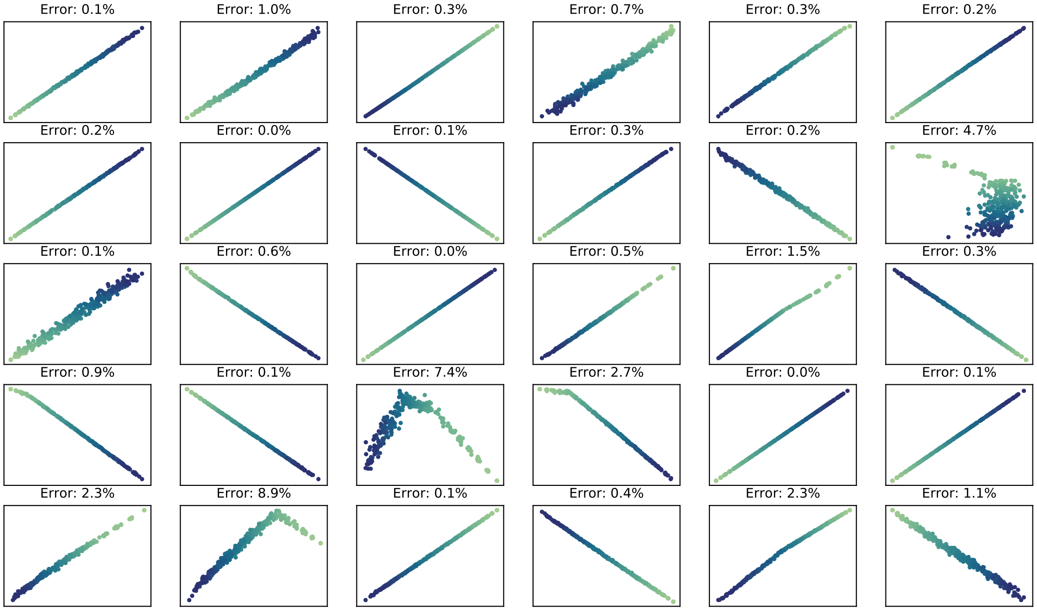

Figure S10: Reproducibility of Jitter Models. We vary the initial seed to determine how reproducible the results are. We find model's trained with the jitter dataset learn mostly linear representations, however, certain seeds do result in large kinks. This indicates that visual variability between scenes will help the model learn the abstraction of number.

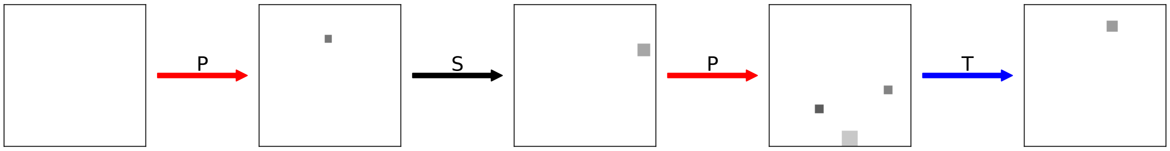

Figure S11: Imprecise Action Datasets. In this dataset, we allow the number of objects taken or placed during an action to be 0-3 (limited by the number of objects in the visual scene). The maximum number of objects is still set to 3. This dataset mimics a situation in which the agent is imprecise with their actions and does not always select one object. The object's size and contrast are randomized between actions (like in dataset B).

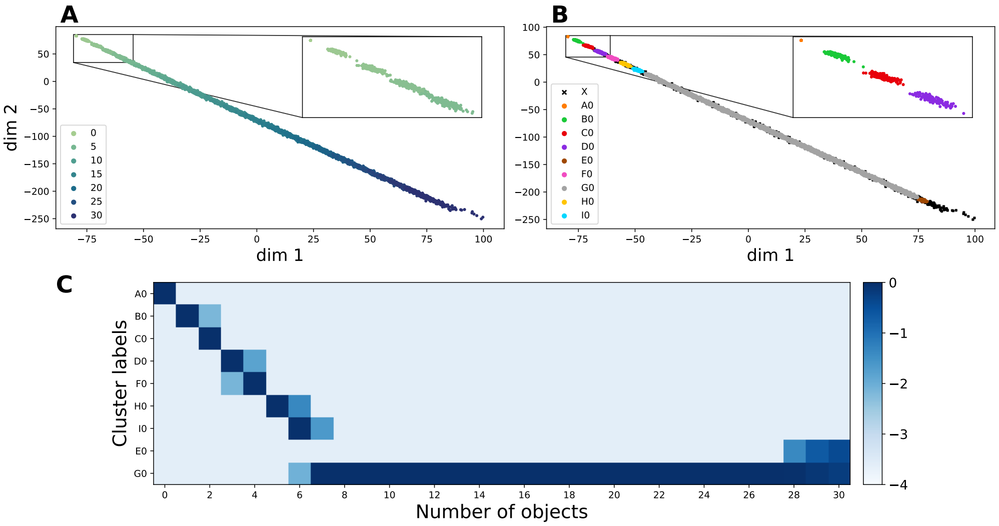

Figure S12: Properties of Imprecise Action Models. We find that the important properties of the Model B representation arise with Imprecise Action Models. The model representations are linear, monotonic, with the early numbers easily separable. However, the separability of the early clusters is rougher than with precise action sizes. We set the minimum cluster size to 50 (HDBSCAN), in order to produce the most concise plots. Note the Imprecise Action Model representations are more sensitive to minimum cluster size.

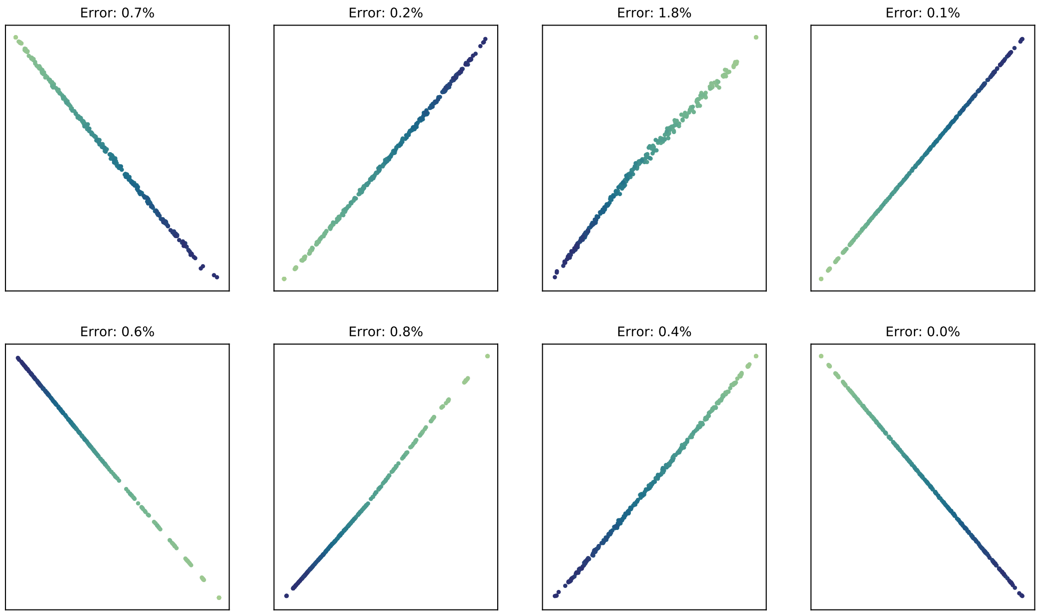

Figure S13: Reproducibility of Imprecise Action Models. We vary the initial seed to determine how reproducible the results are. We find model's trained with the imprecise action sizes learn mostly linear representations.

B Dataset Statistics

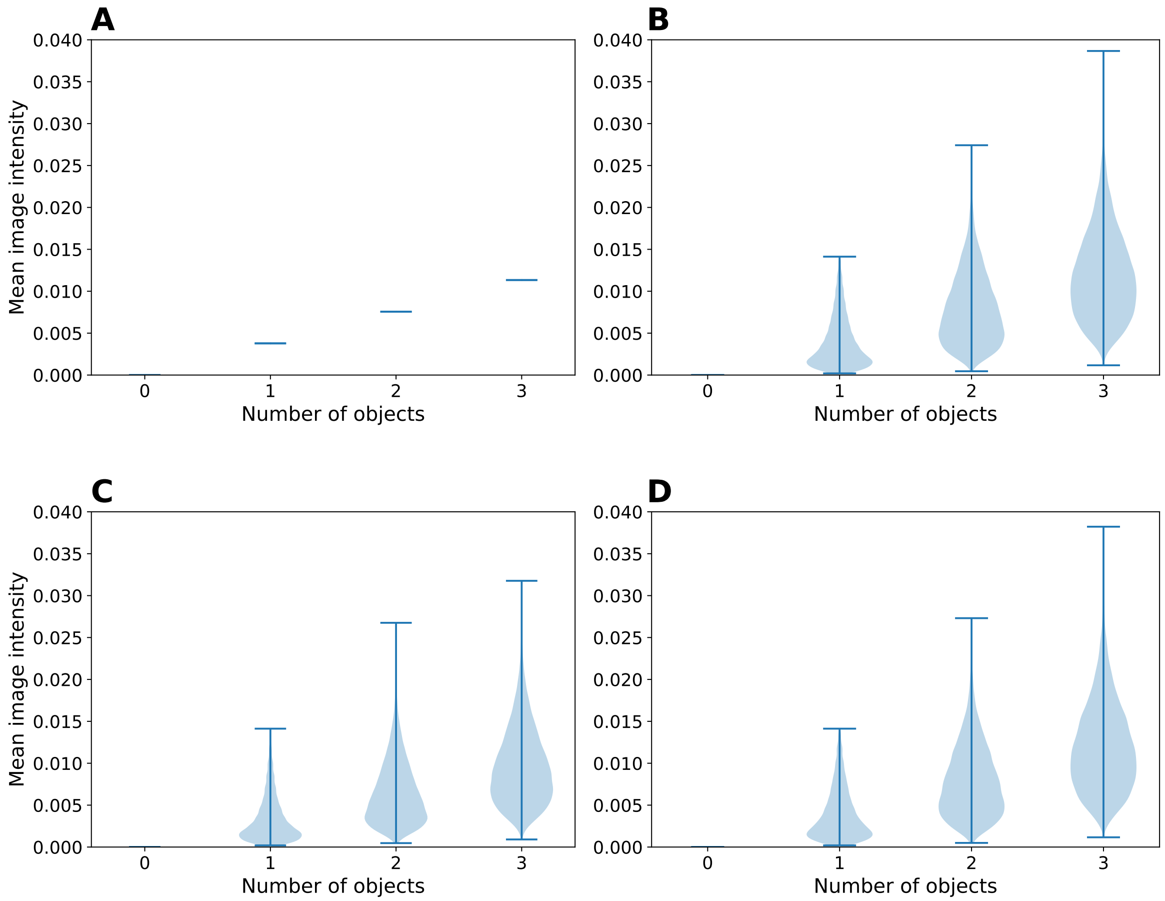

Figure S14: Training set statistics. (A) In dataset A (Fig. 2A) objects have the same size and contrast. Thus, the number of objects predicts the mean image intensity and vice-versa. **(B)** Objects in dataset B (Fig. 2B) have variable sizes and variable contrast, thus mean image intensity is not sufficient to predict the number of objects. **(C)** Objects in the jitter datasets (Fig. S8) have a restricted, but variable size and contrast. We see the image statistics are similar to that of dataset B, but have a smaller amount of variability. **(D)** Objects in the imprecise actions datasets (Fig. S8) have random numbers of objects manipulated in an action. We see the image statistics are effectively the same as that of dataset B.

C Network Details

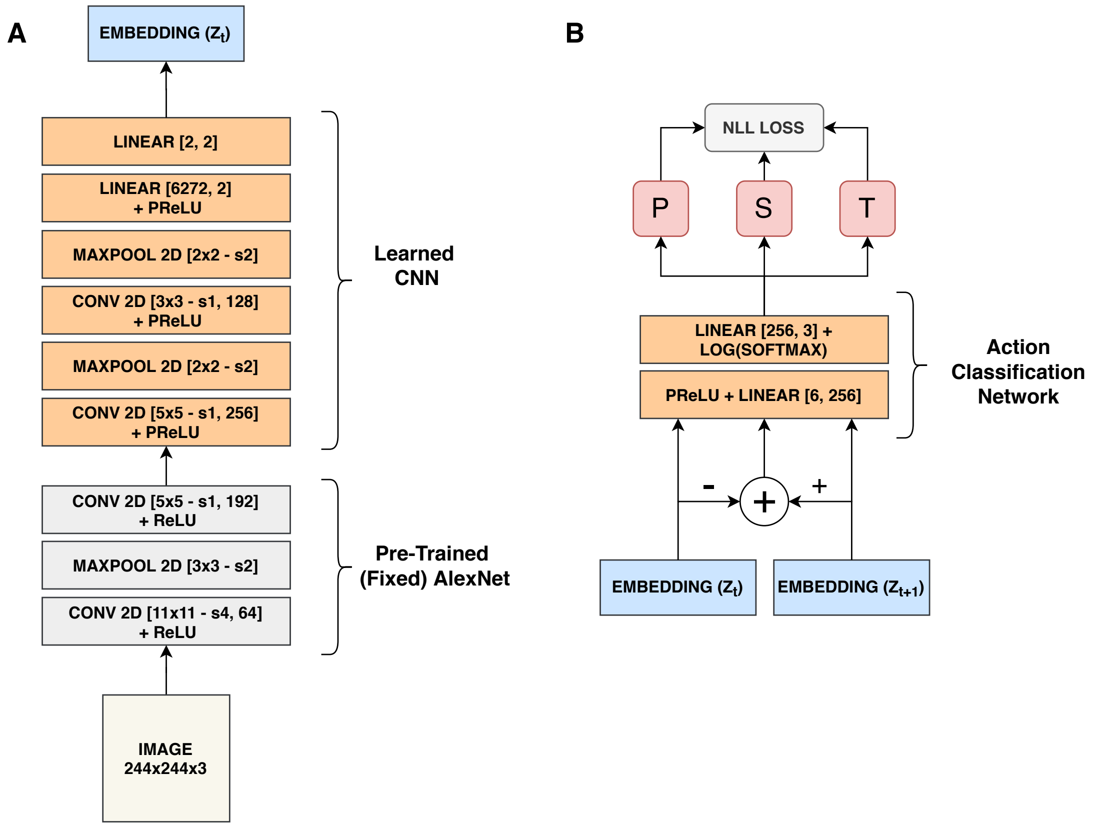

Figure S15: Detailed diagram of the network structure.
(A) The feature extraction / embedding network. The gray layers are pre-trained on ImageNet [51, 27] and remain fixed throughout the course of training. The orange layers are randomly seeded and trained simultaneously with the classifier in (B). The details of the layer are described within the brackets. For example, [11x11 - s4, 64] is an 11x11 kernel with a stride of 4 and 64 filters. During a training step, the embedding network accepts an image (x_t) of the visual scene and generates a lower-dimensional feature embedding (z_t) of the visual scene. An action: (P), (T), or (S) modifies the visual scene and the "after" image (x_{t+1}) is passed through the embedding network as well. The outputs of the embedding network, (z_t) and (z_{t+1}) are treated as inputs to the action classification network. The shared embedding network is trained together with the classifier (B), in a Siamese configuration. **(B)** The action classification network is a 2-layer classifier network and is composed of two fully connected layers with a log-softmax transformation on the output. The input is the representation of the visual scene before and after an action is performed. The negative log-likelihood (NLL) loss function is used to train both the action classification network and the embedding network simultaneously. An overview of the entire training paradigm is shown in Figure 1.
